# Lip Augmentation Using Post-Auricular Fibroareolar Tissue as a New Autogenous Filler

**DOI:** 10.29252/wjps.9.3.274

**Published:** 2020-09

**Authors:** Behrouz Barati, Fatemeh Jahanshahi, Mahboobe Asadi

**Affiliations:** 1 Department of Otolaryngology, Taleghani Hospital, Shahid Beheshti University of Medical Sciences, Tehran, Iran;; 2 Student Research Committee, Faculty of Medicine, Iran University of Medical Sciences, Tehran, Iran

**Keywords:** Lip, Augmentation, Height, Projection, Fibroareolar tissue

## Abstract

**BACKGROUND:**

Augmentation of facial components is an acceptable approach in facial aesthetics. The best filler material and the optimal technique for facial soft tissue augmentation still remain to be determined. This study has assessed the lip augmentation using post-auricular fibroareolar tissue as a new autogenous filler.

**METHODS:**

This prospective study enrolled patients who were candidate for lip augmentation. Loose fibroareolar tissue was harvested from post-auricular region and was inserted in the prepared lip pocket. Standard digital photography was used for lip analysis in each patient before and 6 months after surgery (the height and projection of the upper lip and lower lip).

**RESULTS:**

At a mean follow-up period of 6 months, average upper and lower lip height increased from 5.27 to 8.72 mm (*p*<0.001), and the average upper and lower lip projection increased from 3.97 to 7.75 mm (*p<0*.001).

**CONCLUSION:**

Our technique was minimally invasive and a safe method, in which post-auricular loose fibroareolar tissue was applied for lip augmentation with minimum donor site morbidity and long-term results. On the follow-up, all patients were satisfied with the results and no patients required any revision operation. Moreover, the complication rate was negligible.

## INTRODUCTION

Lips as a major component of the inferior part of the face play a prominent role in attractiveness and youthful appearance. Therefore, finding a safe and permanent procedure with transient side effects, low risk of failure and with natural results to create full lips is still challenging for cosmetic surgeons.^1^^-^^3^ Generally, augmentation of facial components is an acceptable approach in cosmetic procedures, which is extended to the lips. Various methods such as injection and surgery are employed for lips augmentation. The injection method has permanent or temporary results depending on injecting substance. For instance, injecting silicone or polymethacrylate is used for permanent augmentation, while injecting collagen or hyaluronic acid (HA) is for temporary augmentation. The surgery method relies on transferring auto-grafts such as sternocleidomastoid muscle, fat, dermis temporal fascia, and fascialat.^1^^,^^4^^,^^5^

Although injection of fillers as an applicable method is still used, its numerous side effects such as vascular obstruction can result in dermal necrosis or obstruction and cause cerebral ischemia or blindness, local pain, swelling, redness, bacterial infection, long term scar, asymmetry, reaction at injection site, artificial appearance, absorption of fillers and formation of nodules and granulomas in lips. So researchers attempted to develop a permanent method for lip augmentation to have minimum drawbacks too**.**^6^^-^^8^

Lip augmentation candidates are mostly young women who have very thin lips, elderly women who lose their lips fullness as a result of aging, patients who have congenital lip malformation such as cleft, and patients who complain from lip asymmetry or proportionality of upper and lower lips.^3^ Patients’ dissatisfaction is mostly because of the vertical height of either the upper or lower lip or both, insufficient projection of the upper lip, inadequate definition of the contour of the Cupid’s bow, or insubstantial labial volume.^9^

Several materials have been utilized to enhance and contour lips, including alloplastic and autogenous substances. The best filler material and the optimal technique for facial soft tissue augmentation remain to be determined. The ideal technique provides the best aesthetic long-term result, the lowest complication rate and the lowest cost.^10^^,^^11^ We reviewed patients who underwent lip augmentation with post-auricular loose fibroareolar tissue to evaluate this tissue as a safe and effective means for lip augmentation.

## MATERIALS AND METHODS

From September 2012 to October 2013 in a prospective study, the population of patients who presented to the Department of Otolaryngology and Head and Neck Surgery at the Shahid Beheshti University of Medical Sciences (Tehran, Iran) were enrolled. Eligible patients for inclusion in the study were interest in upper and lower lip augmentation. A written informed consent was obtained from all patients for grafting surgical procedure. The study was approved by the Scientific and Ethics Committee of Shahid Beheshti University of Medical Sciences with reference number of IR.SBMU.MSP.REC.1393.113, and human rights were respected in accordance with the Helsinki Declaration. 

**Fig. 1:**
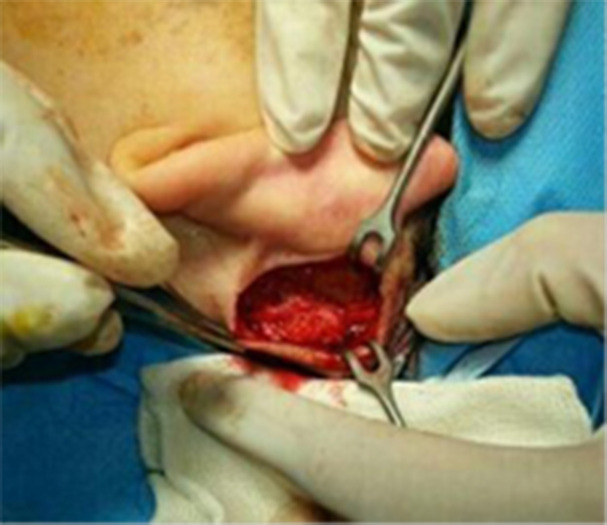
Harvesting fibroareolar tissue from post-auricular region

The exclusion criteria included any previous lip augmentation via injectable and/or surgical fillers in the lips, perioral skin resurfacing and any history of esthetic procedures of the nose, jaws, or anterior teeth. All surgeries were performed by a single surgeon. The graft was harvested from the post auricular loose fibro areolar tissue via post auricular approach. After intravenous sedation or general anesthesia, local anesthesia was accomplished through 5 mL of 2% lidocaine and 1:100,000 epinephrine. then a C-shape incision was made by the number 15 scalpel blade in the postauricular sulcus (Figure 1).

**Fig. 2:**
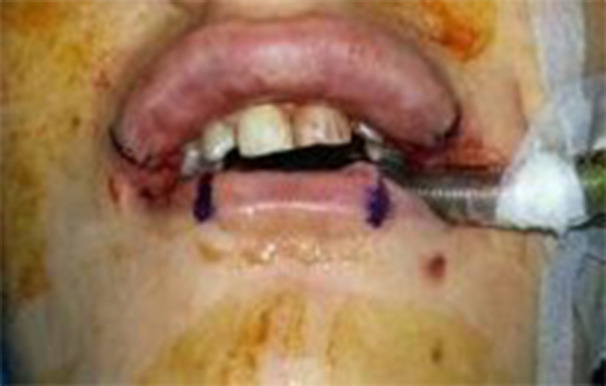
Lower lip marking before augmentation

After the dissection of skin and subcutaneous tissue in the post-auricular region, fibrous tissue, superficial to the periosteum and musculature, was exposed and dissected precisely. A 6-8 mm wide graft strip was prepared from loose fibroareolar tissue according to the length of the lip when the mouth was open. The graft was harvested 5-6 mm more than the measured length of the lip to have excess graft, if a need was arisen during operation. Harvesting areas were closed primarily in 2 layers using 3-0 vicryl sutures in a deep plane and with 5-0 nylon interrupted single sutures on the skin surface. About 2-3 mm incisions were made using scalpel blade number 15 across the upper and lower lip, 4 mm medial to the each oral commissures on the mucosal surface (Figure 2).

**Fig. 3:**
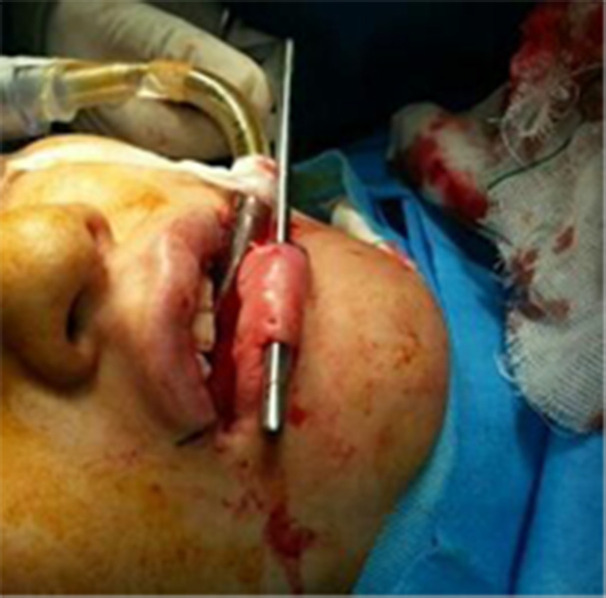
Insertion of cannula at the lower lip

A cannula was inserted and guided toward the other side. The feat tunnel was made with a blunt dissection within the superficial orbicularis oris muscle to avoid invading superior and inferior labial artery. However, caring the vascular supply was more noticeable in injectable fillers and attention was paid in any augmentation procedure. This was repeated several times using a larger cannula to create a proper pocket for the graft strip to be placed (Figure 3).

**Fig. 4:**
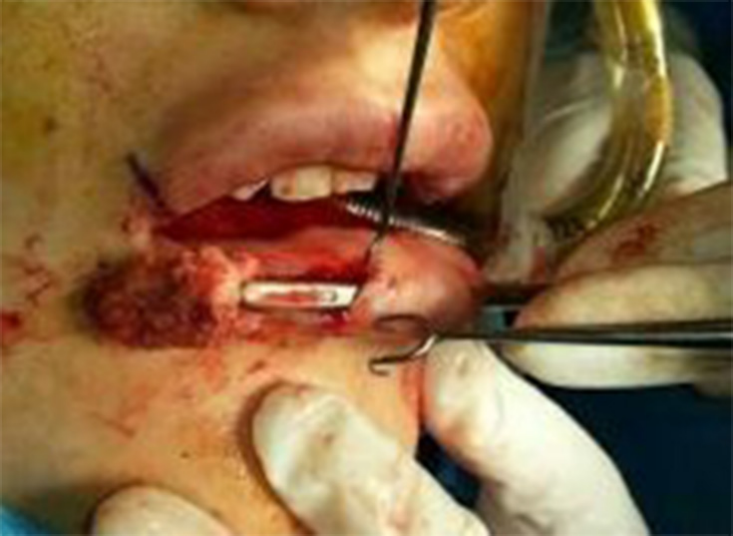
The graft was placed into the prepared tunnel

Afterwards, the graft strip which was cleaned from the muscles and the fat was tailored to an appropriate size and brought into the prepared tunnel by tendon forceps (Figure 4). Its location was adjusted by the hand, once graft placement was satisfactory and the incisions were closed with 5-0 nylon sutures in a precise way to secure the graft against suturing with incisions. 

**Fig. 5:**
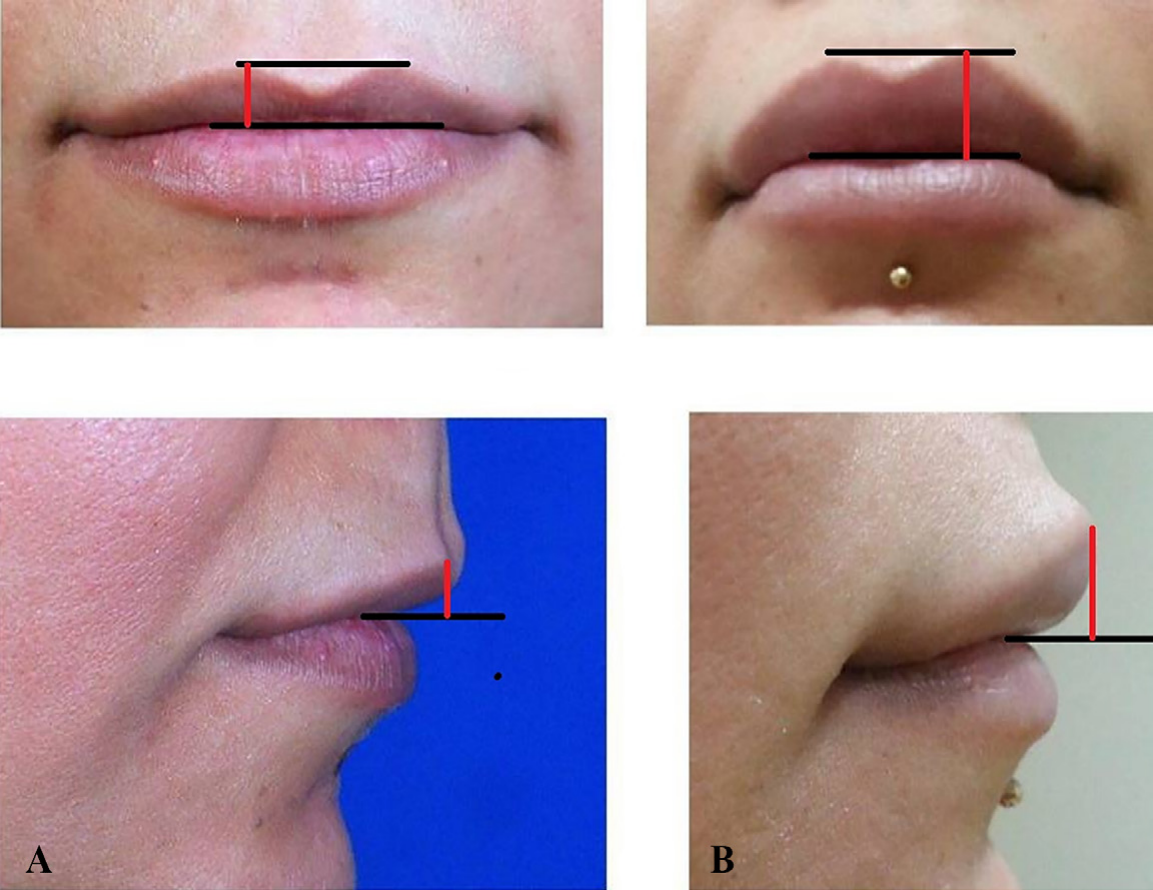
A: Photos on the left: Preoperative upper-lip height. The value of height is indicated in red line. B: Photos on the right: Post operative upper-lip height, 6 months after surgery

All patients received oral antibiotics 1 hour before the procedure and for 2 days post-operation.Depending on patients’ skin type, usually 4 days post-operation, the sutures were removed. The patients were informed that their lips would appear overly corrected because of postoperative swelling in early postoperative period. This swelling subsided gradually to achieve the accepted size and shape. For each patient, a standard digital photo was used for the analysis in lips. The frontal view was used to measure the height of the upper lip and the lower lip (in mm) in the middle of the cupid’s bow. The lip projection was measured on a lateral view as point of maximum protrusion of the vermilion (in mm), perpendicular from a vertical line connecting the base of the columella and to the fold demarcating lower lip and chin. The photographs were taken pre-operatively and 6 months post-operatively (Figure 5). 

The upper and lower lips projection and height were measured before and 6 months after grafting by using Adobe Photoshop 5.0 (Adobe System, Inc., San Jose, CA) and were compared with appropriate statistical analysis using SPSS software (Version 11.5, SPSS Inc., Chicago, IL, USA). Paired sample tests were used for comparison and a p value less than 0.05 was considered statistically significant.

## RESULTS

Ten adult women with the age range from 22 to 38 years old participated in this study. The average age of patients was (30.35±5.34 SD). After six-month follow-up, the height and projection of the upper and lower lips increased from 5.27 to 8.72 mm (*p*<0.001) and from 3.97 to 7.75 mm (*p<0*.001), respectively (Table 1). Before the surgery, the minimum and maximum projections of the upper lips were 2.5 mm and 7 mm, respectively (mean: 4.20 mm). However, 6 months after surgery, the minimum and maximum projection of the lips increased to 5.5 mm and 11.5 mm, respectively (mean: 7.95 mm). The minimum and maximum height of the lips were 3.5 mm and 8 mm, respectively (mean: 5.3 mm), while post-augmentation, the minimum and maximum height of the lips were 6 mm and 12 mm, respectively (mean: 8.8 mm) (Table 1).

**Table 1:** Minimum and maximum lip projection before and after surgery

**Variable**	**Location**	**Time**	**Minimum**	**Maximum**	**Average**	**Standard deviation**	**P value**
Projection	Upper lip	Before surgery	2.5	7	4.20	1.47	<0.001
		6 months after surgery	5.50	11.50	7.95	2.11	
	Lower lip	Before surgery	2	6	3.75	1.29	<0.001
		6 months after surgery	5	11.50	7.55	2.52	
	Both lips	Before surgery	2	7	3.97	1.37	<0.001
		6 months after surgery	5	11.50	7.75	2.27	
Height	Upper lip	Before surgery	3.50	8	5.30	1.47	<0.001
		6 months after surgery	6	12	8.80	2.05	
	Lower lip	Before surgery	4	9	5.25	1.78	<0.001
		6 months after surgery	6	12	8.65	1.98	
	Both lips	Before surgery	3.50	9	5.27	1.59	<0.001
		6 months after surgery	6	12	8.72	1.97	

Before augmentation surgery, the minimum and maximum projections of the lower lip were 2 mm and 6 mm, respectively (mean: 3.75 mm). After augmentation surgery, the values increased to 5 mm and 11.5 mm, respectively (mean: 7.55 mm). Pre-operatively, the minimum and maximum height of the lips was 4 mm and 9 mm, respectively (mean: 5.25). Six months after surgery, the minimum and maximum height of the lips were 6 mm and 12 mm, respectively (mean: 8.65 mm) (Table 1). Before surgery, the minimum and maximum projections of both lips were 2 mm and 7 mm, respectively (mean: 3.97 mm), while 6 months post-operation, the minimum and maximum projections of the lips were 5 mm and 11.5 mm, respectively (mean: 7.75 mm) (Table 1).

**Fig. 6:**
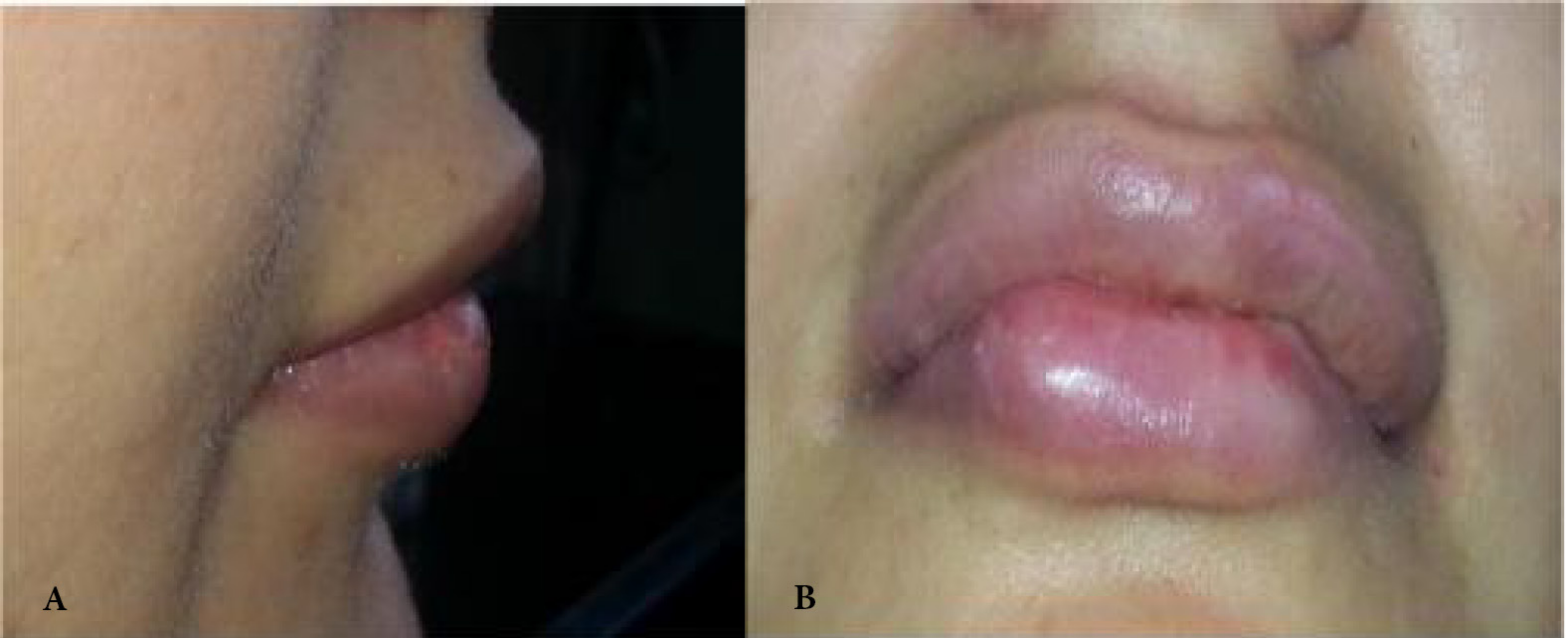
(A): Lateral view show lip edema 10 days after surgery. (B): Frontal view

Preoperatively, the minimum and maximum heights of the lips were 3.5 mm and 9 mm, respectively (mean: 5.27 mm). Six months after augmentation surgery, the minimum and maximum height of the lips were 6 mm and 12 mm, respectively (mean: 8. 72 mm). After lip augmentation surgery, the mean height of the lips increased from 5.27 to 8.72 mm. The investigation on the upper and lower lips individually provided the same results; upper lip and its projection increased from 3.97 to 7.75 mm, respectively. The increase in these 2 values was statistically significant (*p*<0 .001 for the 2 comparisons). No complications such as hematoma, ecchymosis, infection, or neurosensory disturbances were observed. Except temporary hypoesthesia of donor site in 2 cases, no more complications were observed in other donor regions. In some patients, the only concern was lip edema that resolved after 1 to 2 weeks (Figure 6).

## DISCUSSION

The fullness of lips plays a significant role in individuals’ attractiveness and beauty. However, the excess fullness of lips leads to the loss of proportion of the face components and has negative impacts on beauty in addition to hindering lips’ performance, such as speaking and eating. Therefore, cosmetic procedure Results should be aligned with creating proportion between face components.^12^ Congenital diseases such as cleft adversely affect the shape of lips. Additionally, aging can alter cupids bow and vermillion. Moreover, muscle atrophy as a result of aging can cause a decrease in the lip volume and the lip projection. The aging process can be accelerated by sunlight. In addition to natural changes, smoking can make changes in lip muscles and its color. All these reasons make patients as a lip augmentation candidate.^3^

Lip augmentation appeals to a wide range of people to enhance the thickness of lips, and to correct lips deficiencies or senile hypotrophy.^13^ Both vertical height and lip projection in frontal and lateral views should be considered in lip augmentation procedure. The ideal augmentation materials with certain qualities and reproducible results need to be biocompatible, nontoxic, inert, easy to use, and inexpensive.^5^^,^^14^

Generally, there are two basic methods currently being adopted in lip augmentation procedures; one is non-filing and the other one is filing. Direct lip lifting technique, a non-filing method, is performed through direct incision of vermilion border of the upper lip and the lower lip and vertically can extend them upward and downward, respectively, to increase their height. Another technique in this group is indirect lip lifting in which a sub-nasal incision is made and the vermilion border of the upper lip is indirectly lifted by excising the horn-shaped under nose skin.^15^

In the first technique, the incision is made along the whole lip border circumferentially; while in the second, the sub-nasal sill is incised. In these two techniques, it is possible to simultaneously enhance the lip using the excised tissue. In the third group of non-filing methods, corners of the mouth are simply lifted. The skin of the upper commissure of both sides of the lip is excised and the corners are lifted upward. In this way, the age-related changes of the lips are modified and a younger looking shape is created. The fourth technique is called V-incision and Y-closure that leads to lip protrusion. Perhaps the interesting advantage of non-filing techniques is their permanency, but they carry a practical limitation.^15^

These techniques are not applicable to subjects with insufficient volume of lip tissue. This highlights the prominence of lip filing methods such as injection and surgical techniques. With injection methods, the viability of the augmented lip varies enormously, depending on the used material. Hyaluronic acid, collagen, elastin, acellular matrix are materials that offer a transient effect, to name a few. Clearly, these non-invasive provisionally-effective materials need restorative reinjections. Autologous fat tissue can also be obtained through abdominal liposuction, buccal fat pad or presacral liposuction and be utilized. Despite the transitory effect, the fact that the easily-obtained fat tissue, as an autologous, causes no severe foreign body reaction makes it an interesting surrogate.^16^

Agarose and dextran are other injection materials for lip enhancing. To date, various materials have been introduced and utilized for lip enhancing purposes that some of them create more durable effects such as silica and polymethacrylate. However, hyaluronic acid is the most popular filler material and unlike many of them remained the most convenient material for use. Surgical methods were shown to produce more viable results. In these methods, a dermal graft obtained from direct lip lift (DLL); indirect lip lift (ILL) and/or an excised upper eyelid through blepharoplasty can be exploited to create the augmented shape.^16^

Sources including fascia lata, superficial musculoaponeurotic system (SMAS) which is harvested during rhytidoplasty, temporalis fascia, gala and subgala grafts can provide autologous fascia. Tendons are a superb material source for this purpose, as well. There are reports of using palmarislongus tendon which 10% of people naturally do not share. Moreover, lip enhancement techniques can benefit from muscle-obtained grafts and some literature offer promising findings on latissimus dorsi, orbicularis which is harvested during upper eyelid blepharoplasty, sternocleidomastoid muscle (SCM) graft which is developed through rhytidoplasty and buccinators muscle; which is harvested cheek-lip flap.^16^

The side effects associated with lip augmentation processes vary between different techniques; however, they are normally temporary and brief. Generally, these interim side effects range from post-operation swelling and edema to hemostatic disorders which might appear during and or after the surgery and include ecchymosis, bleeding, and bruising. Other possible side effects are infection, abscess, pain and hypoesthesia. Besides, there are some unexpected complications which may occur in the form of lip stiffness and abnormal sensation of material granules, which is associated with the use of muscular grafts and nodule and granuloma formation which might happen as a result of hyaluronic acid injection.^15^^,^^16^

In the present study, the key aspect of the procedure was achieving permanent results for significant increase in the height and projection of the lips. The observed side effects were limited to swelling and edema, though they reduced by applying ice pack or cold compress. In case of an unexpected long lasted swelling or edema, tapered steroid could be administered, although none of the cases in the present study needed such drastic measure. Fortunately, no sign of stiffness was observed in the subjects, yet as a preventive measure, lip exercise could be started. Additionally, it was noticed that the smile and speaking shows of the subjects were cosmetically symmetric. 

In surgical methods, harvesting a graft with minimal damage and side effects is very significant. To prevent foreign body reactions, using auto-grafts is suggested. Loose fibroareolar tissue can be harvested easily via a relatively small incision in post-auricular region. Precise incisions with special attention to hair follicles and tension-free wound closure result in negligible donor-site complications and reduce the risk of visible and hypertrophic scars. Follow-up has shown that this technique results in clinically significant improvement in the mean projection and height of the lips with predictable and long-term results. All patients had normal lips sensation and were satisfied with their surgical results. The limitations of this technique may include limited graft material availability and the difficulty of inserting larger grafts. In a larger group of patients with longer follow-up, comparing of this technique to other augmentation methods confirmed the preliminary findings.

## CONCLUSION

Our technique was minimally invasive and a safe method, in which post-auricular loose fibroareolar tissue was applied for lip augmentation with minimum donor site morbidity and long-term results. On the follow-up, all patients were satisfied with the results and no patients required any revision operation. Moreover, the complication rate was negligible.
